# Prodigiosin/celecoxib-loaded into zein/sodium caseinate nanoparticles as a potential therapy for triple negative breast cancer

**DOI:** 10.1038/s41598-023-50531-4

**Published:** 2024-01-02

**Authors:** Wafaa A. Mohamed, Nefertiti A. El-Nekhily, Hoda E. Mahmoud, Ahmed A. Hussein, Sally A. Sabra

**Affiliations:** Department of Biotechnology, Institute of Graduate Studies and Research, Alexandria University, Alexandria, 21526 Egypt

**Keywords:** Biochemistry, Cancer

## Abstract

Nowadays, breast cancer is considered one of the most upsetting malignancies among females. Encapsulation of celecoxib (CXB) and prodigiosin (PDG) into zein/sodium caseinate nanoparticles (NPs) produce homogenous and spherical nanoparticles with good encapsulation efficiencies (EE %) and bioavailability. In vitro cytotoxicity study conducted on human breast cancer MDA-MB-231 cell lines revealed that there was a significant decline in the IC50 for encapsulated drugs when compared to each drug alone or their free combination. In addition, results demonstrated that there is a synergism between CXB and PDG as their combination indices were 0.62251 and 0.15493, respectively. Moreover, results of scratch wound healing assay revealed enhanced antimigratory effect of free drugs and fabricated NPs in comparison to untreated cells. Furthermore, In vitro results manifested that formulated nanoparticles exhibited induction of apoptosis associated with reduced angiogenesis, proliferation, and inflammation. In conclusion, nanoencapsulation of multiple drugs into nanoparticles might be a promising approach to develop new therapies for the managing of triple negative breast cancer.

## Introduction

Globally, Breast cancer is the most frequent cancer among females and the second leading cause of mortality after lung cancer^1^. In 2018, breast cancer caused more than 7000 deaths and more than 100,000 new cases were diagnosed^2^. This disorder is a multifactorial disease in which many risk factors might contribute to its progression^3^. The first line therapy for breast cancer is usually chemotherapy which can be administrated solely or in combination with radiation or surgery^4^. However, chemotherapeutic drugs usually have several drawbacks including; drug resistance, recurrence and diverse systemic negative side effects^5^. Consequently, there is a great demand to develop new therapy regimens with new drugs such as multi-target inhibitors which could be a single drug with multiple inhibitory effects or combinational therapy of more than one drug so as to simultaneously hit more than one pathway all at once^6^.

Celecoxib (CXB); 4-[5-(4-methylphenyl)–3-(trifluoromethyl)-1H-pyrazol-1-yl] benzene sulfonamide, is a well-known anti- inflammatory drug, selective COX-2 inhibitor and non-steroidal anti-inflammatory drug (NSAID)^7^. It is available in the market in an oral dosage form which was initially approved by the FDA and it was launched by Pfizer, Inc. (New York, NY, USA) in 1999^8^. Cyclooxygenase 2 (COX-2) is well-known to be overexpressed in breast cancer cells leading to excessive conversion of arachidonic acid into prostaglandin E2 (PGE2). This reaction causes progression of breast cancer by several mechanisms including; antitumor immunity suppression, induction of proliferation and angiogenesis. COX-2 also plays a critical role in immune-suppression as it increases infiltration of CD8+ in breast cancer tissue^9^. Meanwhile, it was previously reported that inhibition of COX-2 causes induction of apoptosis via activating caspase 3 and suppressing survivin which is an anti-apoptotic protein^10,11^. CXB exerts its antitumor effect on different breast cancer cell lines depending upon COX-2 (the main inflammatory marker) by inhibiting cell growth and blood supply to tumor cells^12^. CXB is also able to inhibit canonical Wnt/β_Catenin pathway and PI3-K/PDK1/Akt pathway which in turn can inhibit cancer cell survival, angiogenesis, proliferation and migration^13,14^. Moreover, CXB can induce apoptosis by both COX-2 dependent and independent pathways^15^. Unfortunately, CXB has several side effects like other NSAID ranging from mild gastrointestinal tract discomfort to cardiac toxicity, lack of efficient tumor targeting, besides being a more potent anticancer agent when utilized as an adjuvant therapy not as a monotherapy^8^.

Prodigiosin (PDG) is a secondary metabolite microbial red pigment which is produced by a limited group of microorganisms including; *Serratia* spp., *Pseudomonas* and *Streptomyces*^16,17^. It is a naturally occurring tri-pyrrylmethene compound which is insoluble in water and soluble in organic solvents such as acetonitrile and DMSO (dimethyl sulfoxide)^18^. It was isolated from *Serratia* species for the first time by Kraft 1902^19^. PDG has four possible anticancer mechanisms as it can cause (1) cell cycle arrest, (2) change in intracellular pH, (3) induction of mitochondrial-mediated apoptosis and (4) cleavage of cancerous cell’s DNA by inhibiting topoisomerase relaxation via DNA intercalation^20^. In addition, this pigment has antifungal, antibacterial, antiproliferative, and immunosuppressive effects^21,22^. All these actions collectively can cause cancer cell death^23^. PDG also inhibits several pathways such as PI3K/AKT/m-TOR and Wnt/β_Catenin pathways causing further inhibition of cell survival, angiogenesis, proliferation and migration^24,25^. PDG is also expected to inhibit COX-2 like celecoxib and rofecoxib, which was proved by an in silico molecular docking study^26^. Although, PDG seems to be a potent antitumor agent in different cell lines, however, its hydrophobicity and extremely low bioavailability was found to hinder its application as an antitumor agent^27^.

Combinational therapy can afford several benefits in cancer therapy such as simultaneous effect on multiple signaling pathways, reducing the required dose from each drug and hence, reducing their possible side effects^28^. Green nanotechnology is the use of natural or biocompatible components in the synthesis of nanocarriers as they have many privileges such as being biodegradable and non-toxic and the fact that they can provide sustained drug release and increased bioavailability^29^. In general, Nanoparticles (NPs) having size range from 1 to 100 nm can be efficiently taken up by tumor cells since tumor cells have unique features including; leaky blood vessels and weak lymphatic drainage which can permit efficient entrance of NPs into cancer cells via a phenomenon known as enhanced permeability and retention (EPR) effect^30,31^.

Zein is a proline rich water insoluble protein extracted from maize, and it is generally recognized as a safe (GRAS) molecule, so it is thought to be suitable for fabrication of nanocarriers that can encapsulate hydrophobic drugs^31^. Zein NPs have been fabricated by several methods like antisolvent precipitation, heat-induced self-assembly and pH-induced self-assembly techniques. However, naked zein NPs are relatively unstable, therefore, they are usually coated by another protein or polysaccharide polymers^32^. Sodium caseinate (Na CAS) is an amphiphilic and stable protein extracted from milk. It is also biocompatible, GRAS and biodegradable so it was previously utilized in coating of zein NPs^33^.

As both drugs; CXB and PDG are poorly soluble in water with low bioavailability, they can be-loaded into NPs in order to improve their aqueous solubility^18,33^. CXB was previously encapsulated into several nanocarriers such as casein NPs, chitosan-fucoidan NPs, polylactide-Co-Glycolide NPs, inulin-based Nano micelles, PEGylated liposomes and solid lipid NPs^33–38^. Due to the hydrophobicity of PDG, it was-loaded into several nanocarriers such as; grafted β-cyclodextrin, chitosan magnetic NPs, chitosan nanospheres, selenium NPs and β-casein coated lactoferrin NPs^39–42^. It was also-loaded in implantable biomedical device made from poly-di-methyl-siloxane (PDMS) which contains thermosensitive Poly(*N*-isopropylacrylamide) and it was also-loaded into hybrid nanofiber made from poly(d,l-lactic-co-glycolic acid) (PLGA) with gelatin or Pluronic F127 for local management of triple negative breast cancer cell line^43,44^.

In this study, bacterial PDG and CXB were co-loaded into zein(core)/Na. caseinate (shell) NPs. Zein NPs were coated with Na caseinate to increase the stability of the formulated zein NPs. Both drugs were used together to hit multiple pathways to overcome drug resistance of triple negative breast cancer. Cytotoxicity of both free and formulated drugs was determined by MTT assay against MDA-MB-231 human breast cancer cells. In addition, the migration of cancer cells after treatment with free or-loaded drugs was assessed by scratch wound healing assay. Moreover, Ki-67 was measured as a proliferative marker using flow cytometry assay. Angiogenesis, inflammation, and apoptosis were also assessed by measuring the angiogenic marker, vascular endothelial growth factor (VEGF); PGE2 was measured as a function of COX-2 and the apoptotic marker; caspase3 by ELISA technique.

## Results

### Production and characterization of prodigiosin from *S. marcescens*

**Figure 1:**
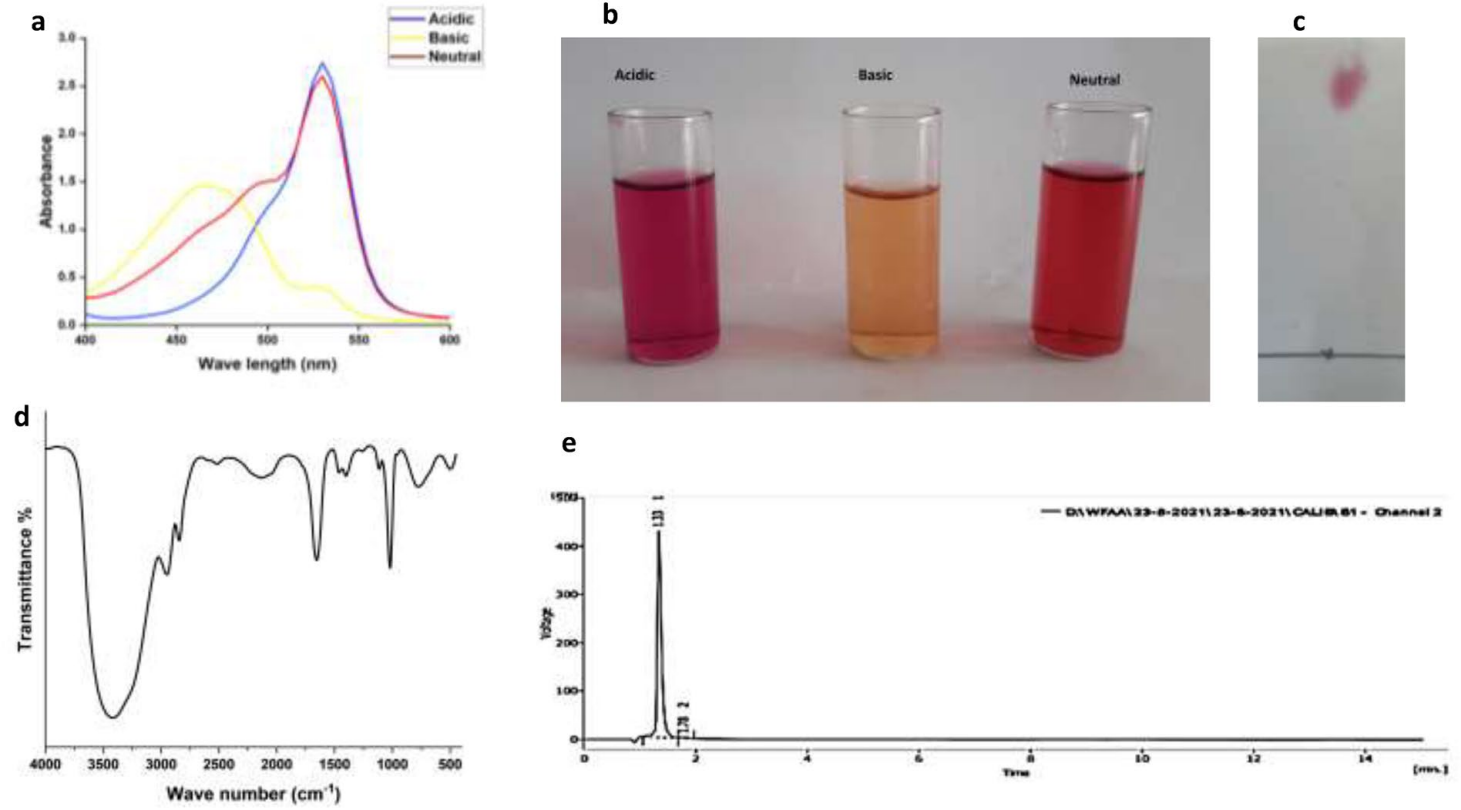
Characterization of the extracted bacterial prodigiosin (PDG). UV–VIS spectrum of PDG (**a**), different coloration of PDG at different pH values (**b**), purified PDG on thin layer chromatography (TLC) plate (**c**), Fourier transform infrared (FTIR) spectrum of PDG (**d**), high performance liquid chromatography (HPLC) spectrum of purified PDG (**e**).

The maximum amount of prodigiosin was achieved on peanut media after 48 h at 28 °C which is consistent with previous research^17,45^. At the end of the extraction, the prodigiosin yield was approximately 250 mg from 1 L of culture medium. The purification of the microbial pigment by gravity column chromatography was about 92%^42,46^. Spectroscopic analysis of PDG showed that it displayed different spectrum and solution color according to the examined pH (Fig. 1a and b). In acidic condition (pH 2), it exhibited pink color with maximum absorbance at 530 nm, while in neutral condition (pH 7), the maximum absorption peak intensity decreased to reach 1%, and the color turned into red. In alkaline condition (pH 9), the maximum absorption was shifted to 465 nm with a yellow color. Furthermore, PDG was also characterized by TLC and it exhibited Retention factor (RF) of 0.9 which was consistent with the RF of standard PDG as previously reported in the literature as shown in (Fig. 1c)^16^.

The extracted pigment showed Fourier transform infrared (FTIR) spectrum with a broad stretching band at 3417 cm^−1^ (–OH & –NH stretching of pyrrole ring), aromatic –CH attached to methylene-O showed a stretching band at 2950 and 2844 cm^−1^, C=, and alkynes showed a weak stretching band at 2130 cm^−1^. A medium stretching band was determined at 1655 cm^−1^ relating to (C=C & C=N bending), a weak bending band at 1460 cm^−1^(C–C aromatic), 1402 cm^−1^ (C–N aromatic), 1113 cm^−1^(C–O or C–N aromatic), 1020 cm^−1^ (C–H_2_ aromatic) and at 777 cm^−1^ (alkyl-C) were also observed. Finger print regions of standard PDG as reported in literature were observed at 1655 cm^−1^ (C=N or C=N) at 1402 cm^−1^ (C–N aromatic), 1113 cm^−1^ (C–O aromatic), 1020 cm^−1^ (C–H2 aromatic) and 777 cm^−1^ (C-alkyl), indicating the presence of (–C–O–C–) linked to aromatic pyrrole ring^47–49^. As shown in Fig. 1d, FTIR spectrum revealed that the extracted pigment contains pyrrole, methylene, alkane, and alkene, which are in accordance with the spectrum of the standard PDG. Moreover, high performance liquid chromatography (HPLC) analysis of PDG confirmed the purity of the extracted pigment showing a single peak with retention time of about 1.3 min (Fig. 1e).

### Preparation of PDG/CXB-loaded zein/ Na CAS NPs

Zein was used as hydrophobic core for encapsulation of CXB/PDG due to their hydrophobicity and low water solubility. Lower water solubility of this protein creates more supersaturation, which increases the rate of nucleation and formation of smaller NPs. Sodium caseinate has good emulsifying and stabilizing properties when it is used as a coating agent in aqueous solution. Caseinate has negative charge and it can adsorb easily onto the surface of positively charged zein NPs resulting in more stable colloidal NPs^50^. Zein/caseinate mass ratio 1:1 was the most optimum formulation as it renders the best encapsulation efficiency (EE%) and particle size (PS). Different ratios showed low EE% and PS^51^.

### Physicochemical characterization of PDG/CXB-loaded zein/ Na CAS NPs

**Figure 2:**
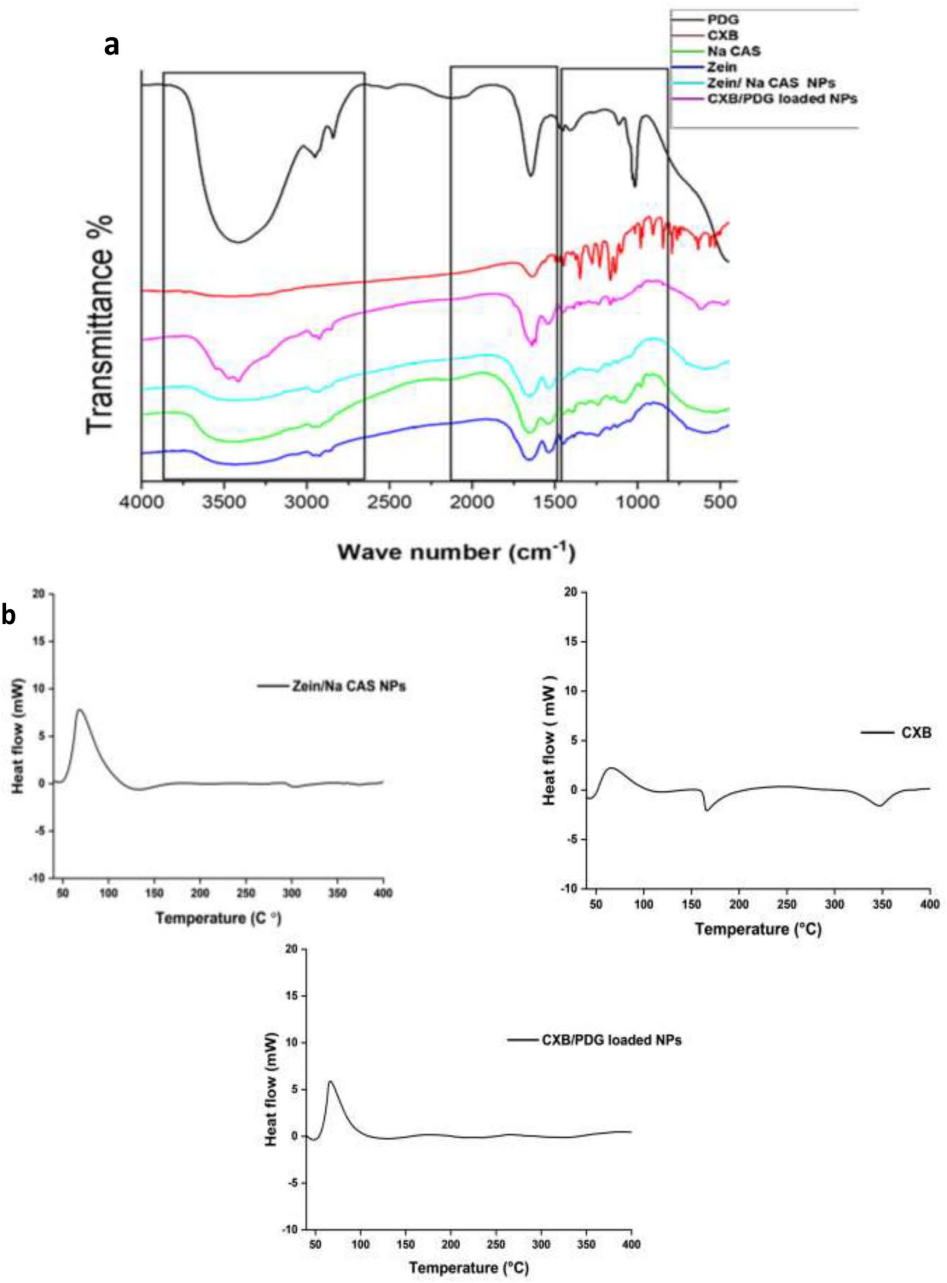
Fourier transform infrared (FTIR) spectra of free prodigiosin (PDG), free celecoxib (CXB), sodium caseinate (Na CAS), zein/Na CAS NPs and CXB/PDG-loaded NPs (**a**), differential scanning calorimetry (DSC) thermogram of free CXB, zein/Na CAS NPs and CXB/PDG-loaded NPs (**b**).

Free proteins and blank zein/Na CAS NPs exhibited in their FTIR spectrum the most obvious peaks of amide I at (1655–1645) cm^−1^ owing to C=O stretching vibration and amide II at (1540–1530) cm^−1^ owing to N–H bending with C–N stretching vibrations. Also, a peak for hydroxyl group was observed at 3451 cm^−1^, CH_2_ bending at 1448 cm^−1^ and stretching of CH_3_ and CH_2_ were located at 2925 and 2963 cm^−1^, respectively. The region between 1000 and 1400 cm^−1^ referred to the side chain of amino acids^31,52^. The reported finger print regions of PDG were observed in the spectrum and they were consistent with our isolated pigment^47–49^. CXB revealed a mild stretching peak of –NH at 3300–3600 cm^−1^, a stretching peak of NH_2_ at 1636 cm^−1^, a stretching peak of S=O at 1348 cm^−1^ and a stretching peak of C–F at 1230 cm^−1^^53^. FTIR spectrum of CXB/PDG-loaded NPs exhibited the major peaks of CXB and PDG which appeared in the range of 3250–3750 cm^−1^. Also, no significant shift was observed in the characteristic peaks of both drugs, while some other peaks disappeared due encapsulation of drugs into the hydrophobic core of the prepared NPs (Fig. 2a)^31,37^.

Thermal behavior of free CXB, blank NPs and CXB/PDG-loaded zein/Na CAS NPs were further analyzed by differential scanning calorimetry (DSC). Upon heating, CXB was converted from an amorphous state into crystalline at 65.95 °C (exothermic peak) and some energy was released (Fig. 2b). As the heating continued, CXB began to melts at 165.41 °C (endothermic peak) and finally decomposes at 349.04 °C (endothermic peak)^54^. Differential scanning calorimetry (DSC) thermogram of CXB/PDG-loaded NPs and blank NPs showed only one exothermic peak at 68 ± 0.5 °C which may refer to crystallization of NPs^55^. Due to encapsulation of both drugs within zein/Na CAS NPs in an amorphous state or as molecular dispersion within the core of the particles, the endothermic peaks of both CXB and PDG in the DSC thermograms were completely absent^31^.

**Figure 3:**
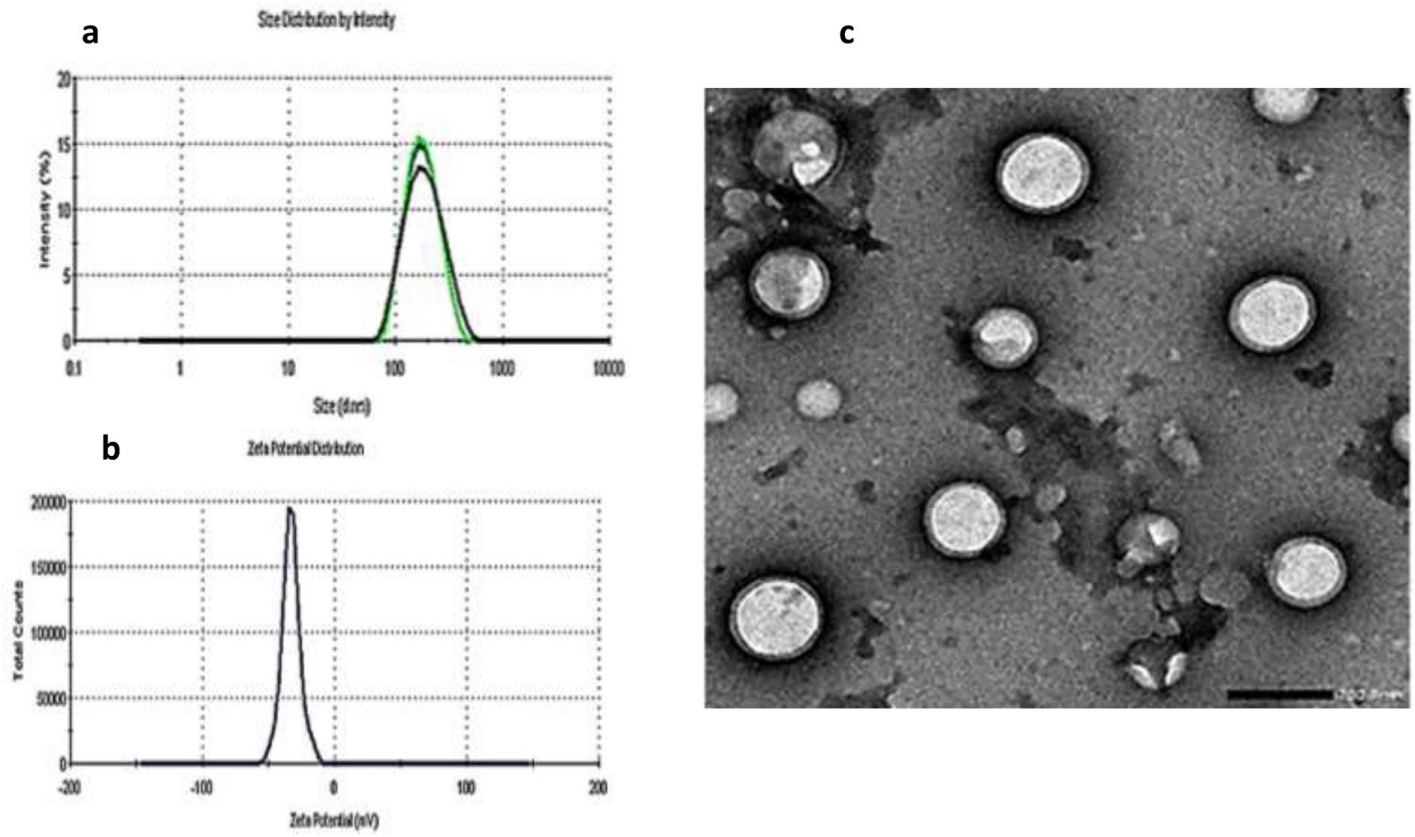
Particle size of celecoxib/prodigiosin-loaded NPs (**a**), Zeta potential (**b**), transmission electron microscope (TEM) micrograph of celecoxib/prodigiosin-loaded NPs; scale bar is 200 nm (**c**).

The average particle size (PS) of blank zein/Na CAS NPs was found to be 144.7 ± 2.696 nm with poly dispersion index (PDI) of about 0.201 ± 0.027 with zeta potential of about − 33.1 mV. Dual loading of PDG and CXB into zein/Na CAS NPs resulted in an increase in the PS to 171.2 ± 3.535 nm and PDI value was 0.142 ± 0.043 without any significant change in zeta potential value (− 34.9 mV), suggesting the stability of the prepared NPs, as for a stable NPs suspension, its zeta potential must be at least 20 mV which can provide sufficient repulsion between NPs and hence can prevent their aggregation (Fig. 3a, b)^56^.

Morphological examination by transmission electron microscope (TEM) revealed that prepared dual drug-loaded NPs were spherical in shape and a diameter of about 151 nm with a characteristic core/shell structure comprised of zein as the hydrophobic core surrounded by hydrophilic Na CAS shell (Fig. 3c). The diameter of NPs measured by TEM was smaller than the hydrodynamic PS (about 171.2 ± 3.535 nm) due to the presence of an outer water shell around the NPs in case of measuring the hydrodynamic PS^57^.

**Figure 4:**
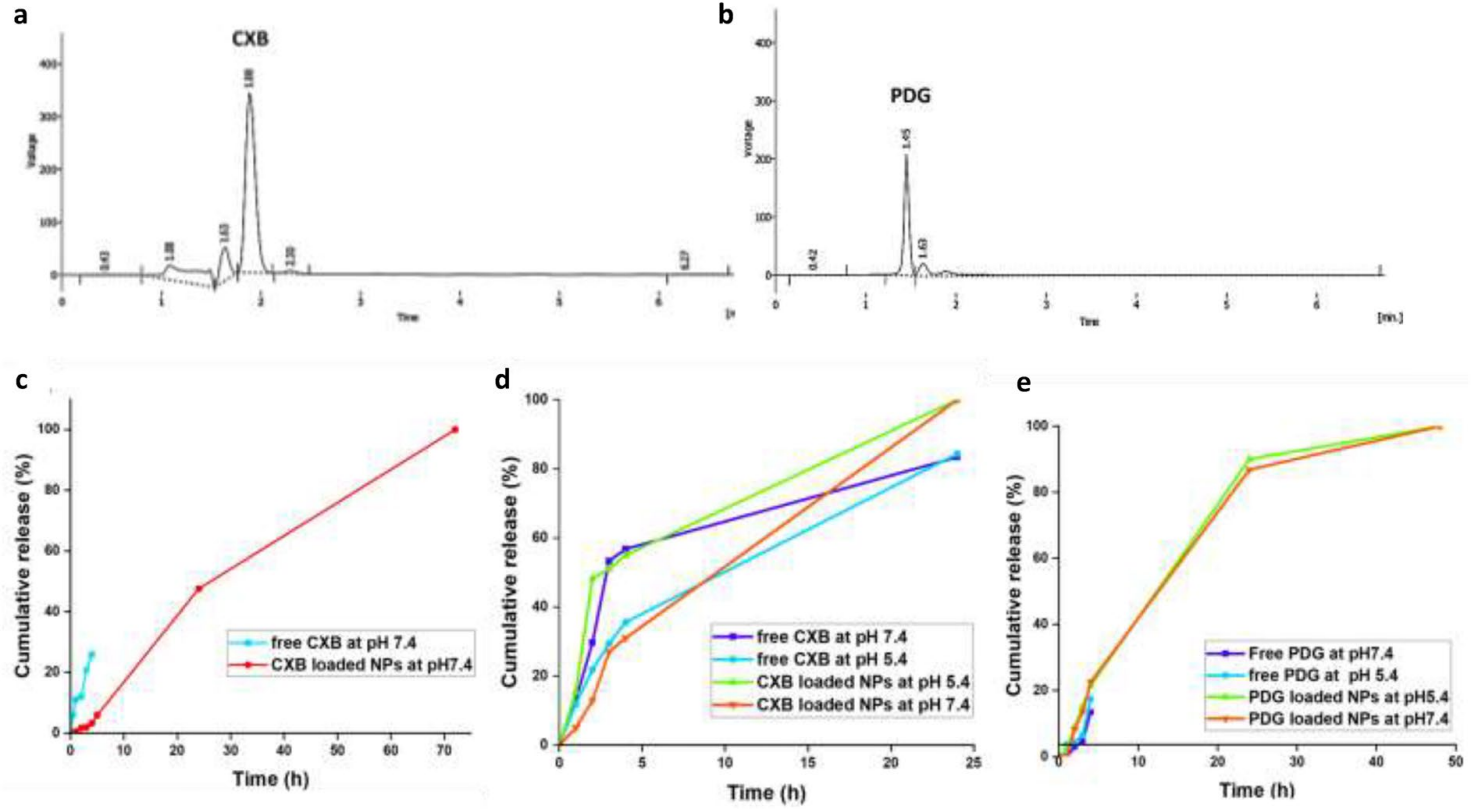
High performance liquid chromatography (HPLC) spectrum of encapsulated celecoxib (CXB) (**a**), prodigiosin (PDG) (**b**), In vitro release profile of CXB in PBS at pH 7 (**c**). CXB in PBS; pH 7.4 and 5.4 with 50% ethanol (**d**), PDG in PBS; pH 7.4 and 5.4 with 50% ethanol (**e**).

Encapsulation efficiency (EE %) of PDG was 82.44% ± 2.05 and CXB was 74.57% ± 2.97. Drug loading (DL) % of PDG and CXB were 3.65% and 3.52%, respectively. Encapsulation of a drug into nanocarrier depends on the drug dose and the nature of both the drug and the utilized polymers included in the synthesis of nanocarrier. As shown in (Fig. 4a, b), the obtained EE% of PDG and CXB were good even though hydrophobic drugs usually have low EE as previously reported^38,40^. Zein is an amphiphilic protein which can form hydrophobic core suitable for encapsulation of hydrophobic drugs^31^. PDG as an anticancer drug is effective at low dose, while CXB requires a higher dose to exert its anticancer effect. Consequently, the obtained EE% and DL% make zein/Na CAS NPs a suitable carrier for co-encapsulation of PDG and CXB^25,58,59^.

#### In vitro drug release

In vitro release profile of free CXB revealed that 25.9% of the free drug was released after 5 h, then it started to precipitate in PBS at pH 7.4 due to its hydrophobicity and low dissolution rate in water^60,61^. While formulated CXB revealed a sustained release profile as only 5.9% was released after 5 h. This small amount of released drug might be due to adsorption of some drug molecules on the outer surface of the NPs^31^. After 24 h, 47.6% of CXB was released and 100% release was achieved after 72 h. Regarding PDG, it did not exhibit any release in PBS buffer even after its supplementation with 3% Tween-80 due to its extreme hydrophobicity As shown in (Fig. 4c)^18,62^. As a result, the release medium was selected to be PBS with 50% ethanol at both pH 7.4 and 5.4. Addition of ethanol into PBS was found to fasten the release of CXB from the NPs with increased release in the acidic medium in comparison to the neutral medium (Fig. 4d). At pH 7.4, about 30% of CXB was released after 4 h but at pH 5.4, 55% was released and almost 100% of encapsulated CXB was released at pH 7.4 and 5.4 after 24 h. In vitro release profile of PDG showed that free PDG was precipitated after 4 h of incubation, while-loaded PDG showed sustained release as only about 23% was released at the first four h, then 90% was released after 24 h followed by 100% release after 48 h. Furthermore, there was no change in the release profile of PDG in different pH as shown in (Fig. 4e).

The hydrodynamic particle size, PDI and zeta potential of dual drug-loaded NPs were measured after 24 h incubation in PBS; pH 7.4 and PBS; pH 5.4 supplemented with 50% ethanol. As shown in supplementary Fig. 1(S1), results revealed that at pH 7.4, there was a decrease in the size of NPs from 171.2 ± 3.535 to 156 ± 7.800. This decline in the PS might be related to release of about 90% of the encapsulated drugs. However, there was slight increase in the PDI value from 0.142 ± 0.043 to 0.352 ± 0.0175, which could be attributed to formation of some aggregates with a percent of about 10% due to presence of ethanol that could cause partial solubilization of zein protein and aggregation sodium caseinate^63,64^. Zeta potential of dual drug-loaded NPs was − 30.2 mV.

At pH 5.4, there was also a decrease in the size from 171.2 ± 3.535 to 137.7 ± 6.885 nm, which might also be due to release of about 90% of the drug. In case of acidic medium, there was a significant increase in the PDI level from 0.142 ± 0.043 to 0.633 ± 0.032(~ 4-fold increase) due to formation of large aggregates with percent of about 25%. High percent of aggregates was formed in acidic medium due to presence of sodium caseinate protein as a coat for the dual drug-loaded NPs, which is reported to have an isoelectric point of about 4.26, which is very close to the pH of the release medium and might induce formation of large aggregates^64,65^. Zeta potential of dual drug-loaded NPs was − 29.8 mV. Moreover, the model of drugs release from prepared nanoformulation was determined by analyzing the results of in vitro release experiment with model-dependent methods. The results are shown in supplementary table 1 and supplementary Figs. 2 and 3.

#### In vitro blood hemolysis

**Figure 5:**
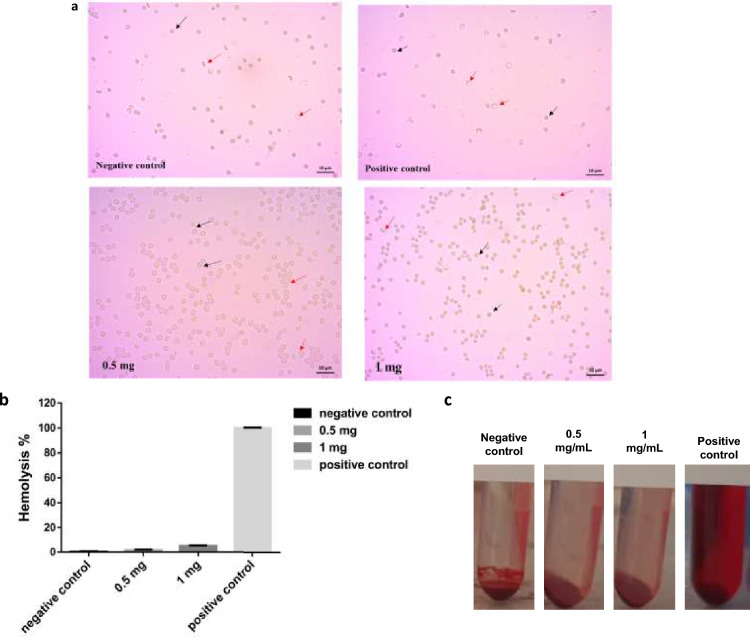
In vitro blood hemolysis of celecoxib/prodigiosin-loaded NPs (CXB/PDG-loaded NPs). Black arrows represent non hemolyzed red blood cells (RBCs) and red arrows represent hemolyzed RBCs; Scale bar is 10 μm (**a**), % of released hemoglobin (**b**), an image showing incubated RBCs with different formulation concentrations (**c**). Results are expressed as mean ± SD.

As shown in Fig. 5, Hemolysis % which caused by the utilized amount of the formulated NPs (0.5 and 1 mg) were 1.5% and 4.9% respectively. Hemolysis was also estimated by examination of the morphology of RBCs under the light microscope. From the results obtained, it was observed that most of RBCs in the positive control group were empty and shrunk with hemoglobin released from the cells. While most RBCs in negative control group and most of the examined concentrations of CXB/PDG-loaded NPs were still maintaining their proper shape with small fraction of hemolyzed cells.

#### In vitro cytotoxicity study

**Figure 6:**
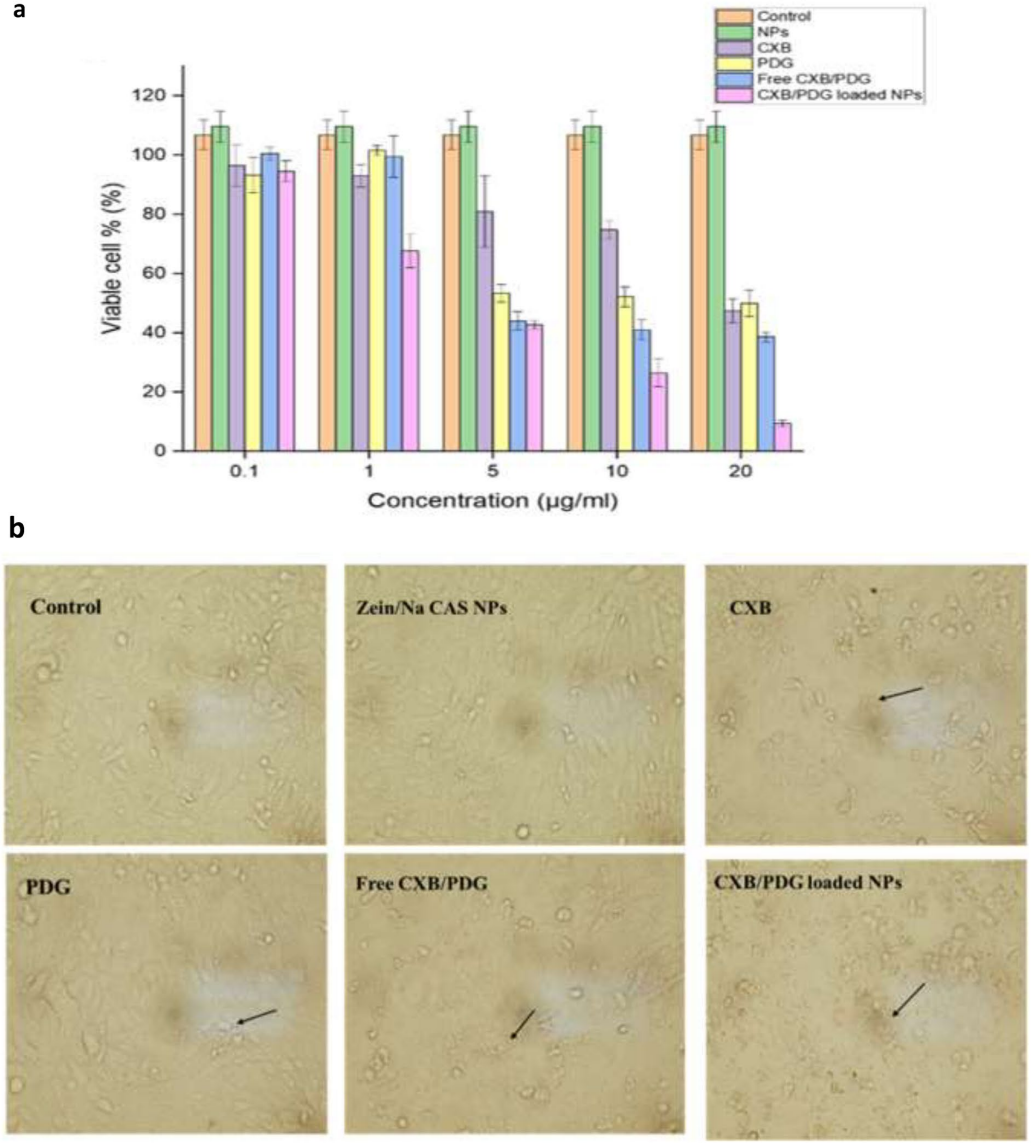
Cellular viability upon treating cells with celecoxib/prodigiosin-loaded NPs (CXB/PDG-loaded NPs) in comparison to free combination or control groups (**a**), morphological changes of MDA-MB-231 cell line by treating with 5 μg/mL of free prodigiosin (PDG), free (CXB), free CXB/PDG, zein/Na CAS NPs and CXB/PDG-loaded NPs in comparison with control group (**b**). Black arrows represent cellular debris. Results are expressed as mean ± SD.

The cytotoxic effect of CXB/PDG-loaded NPs was examined in triple negative breast cancer cell line (MDA-MB-231). The IC_50_ of free PDG was found to be 21.396 μg mL^−1^ and that of free CXB was found to be 92.602 μg mL^−1^, while IC_50_ of free combination drugs (CXB/PDG) was found to be 10.8195 μg mL^−1^ for each drug as shown in Table 1. The inhibitory effect of the free combination was found to be in a dose-dependent manner (Fig. 6a). Encapsulation of PDG and CXB into zein/Na CAS NPs was found to enhance their cytotoxic effect in comparison to free combination by decreasing the percentage of viable cells especially at high doses. For instance, using 20 μg mL^−1^ of either drug in dual-loaded NPs had a cell viability of only 9.47 ± 1% compared to 38.59 ± 1.6% when using a mixture of free drugs at same concentration (free combination). Moreover, blank NPs didn’t demonstrate any cytotoxic effect on MDA-MB-231 cells with a cellular viability % of more than 100 ± 7%.

**Table 1:** Calculated IC_50_ and combination index (CI) by Compusyn software of celecoxib (CXB), prodigiosin (PDG), free CXB/PDG and CXB/PDG-loaded NPs.

Drug/Combo	CI value	Dose PDG (μg mL^−1^)	Dose CXB (μg mL^−1^)
CXB		21.396	
PDG			92.6024
Free CXB/PDG	0.62251	10.8195	10.8195
CXB/PDG-loaded NPs	0.15493	2.69277	2.69277

**Figure 7:**
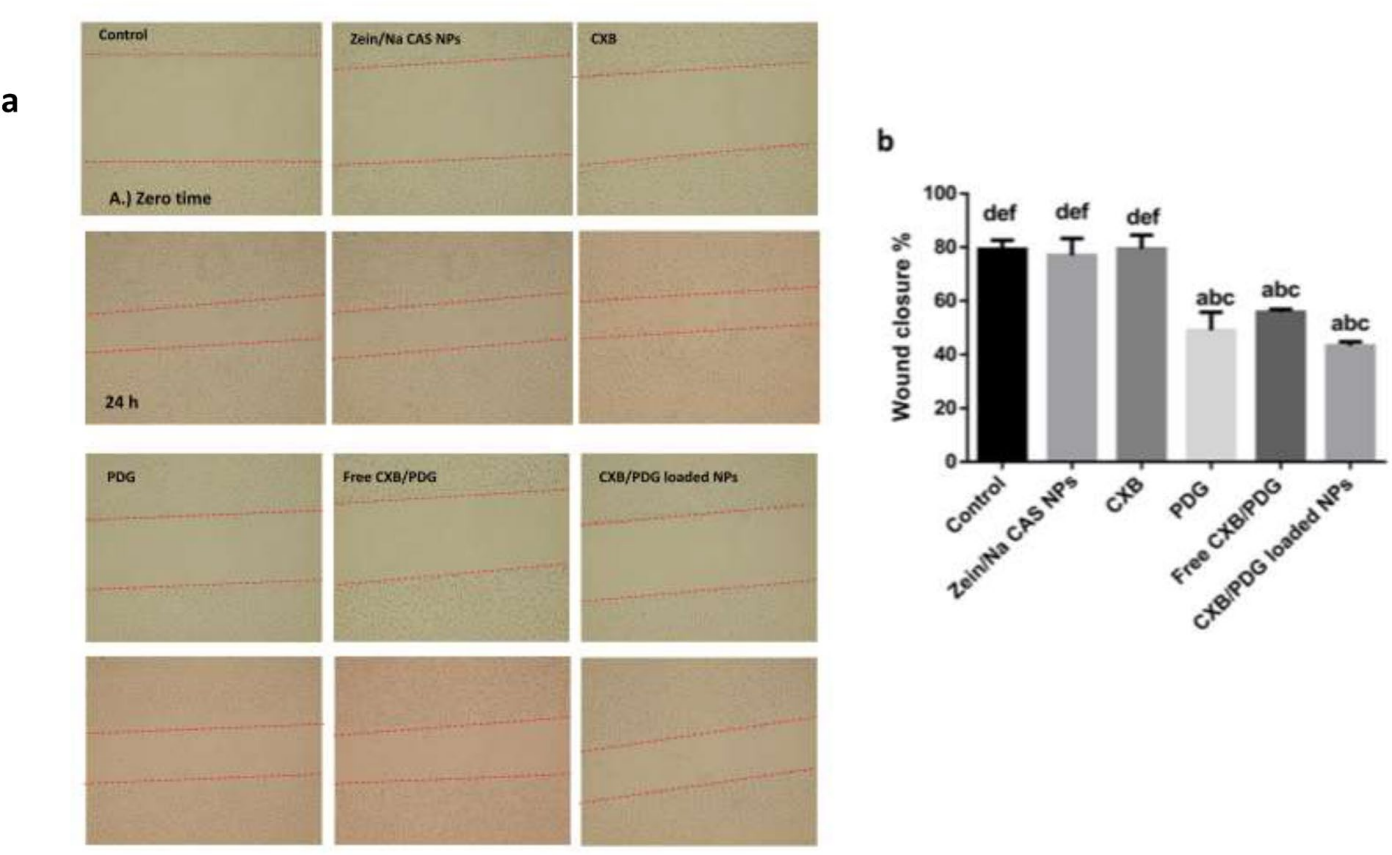
Scratch wound healing assay on MDA-MB-231 cell line at Zero time and after 24h upon incubation with 10 μg of zein/Na CAS NPs, free celecoxib (CXB), free prodigiosin (PDG), free CXB/PDG and CXB/PDG-loaded NPs (**a**), wound closure % of treated and untreated groups (**b**). Values are represented as mean ± SD, n = 3. Statistical analyses were done using one-way ANOVA followed by Post Hoc Test (Tukey). (**a**) P ≤ 0.05 vs control, (**b**) P ≤ 0.05 vs zein/Na CAS NPs, (**c**) P ≤ 0.05 vs free CXB, (**d**) P ≤ 0.05 vs free PDG, (**e**) P ≤ 0.05 vs free CXB/PDG and (**f**) P ≤ 0.05 vs CXB/PDG-loaded NPs.

### Effect of PDG, CXB and their combination on the morphology of MDA-MD-231 cell line

As shown in Fig. 6b, there were some morphological changes and an increase in the amount of apoptotic cells upon cell treatment (especially in case of CXB/PDG-loaded NPs group). Cells were treated with 5 μg/mL of PDG, CXB, free combination, blank NPs and CXB/PDG-loaded NPs. There was an observed large amount of cell debris and apoptotic bodies in CXB/PDG-loaded NPs. There was a great reduction in the dose of CXB and PDG upon treating of cancer cell by their free combination or CXB/PDG-loaded NPs due to a possible synergism between CXB and PDG as the CI of free combined drugs and CXP/PDG-loaded NPs were 0.62251 and 0.15493, respectively.

### Migration assay

The anti-migratory potencies of CXB, PDG, free combination (CXB/PDG), CXB/PDG-loaded NPs and blank zein/Na CAS NPs were estimated by scratch wound healing assay on MDA-MB-231 cell line. The width of the wound was measured at zero time and after 24 h. As shown in Fig. 7a and b, the percentage of wound closure in the control group, zein/Na CAS NPs group and CXB-treated group were 79.22 ± 3.3%, 67.9 ± 6.3% and 79.36 ± 6.7%, respectively, without any significant difference between them. The most pronounced inhibition of cancer cell migration was observed when cells were treated with free PDG, free CXB/PDG and CXB/PDG-loaded NPs group as the percentage of wound closure were 49 ± 6.7%, 55.75 ± 1.1%, 43.2 ± 1.5%, respectively.

### In vitro antitumor efficacy

#### Effect of CXB, PDG and their combination on Ki-67

**Figure 8:**
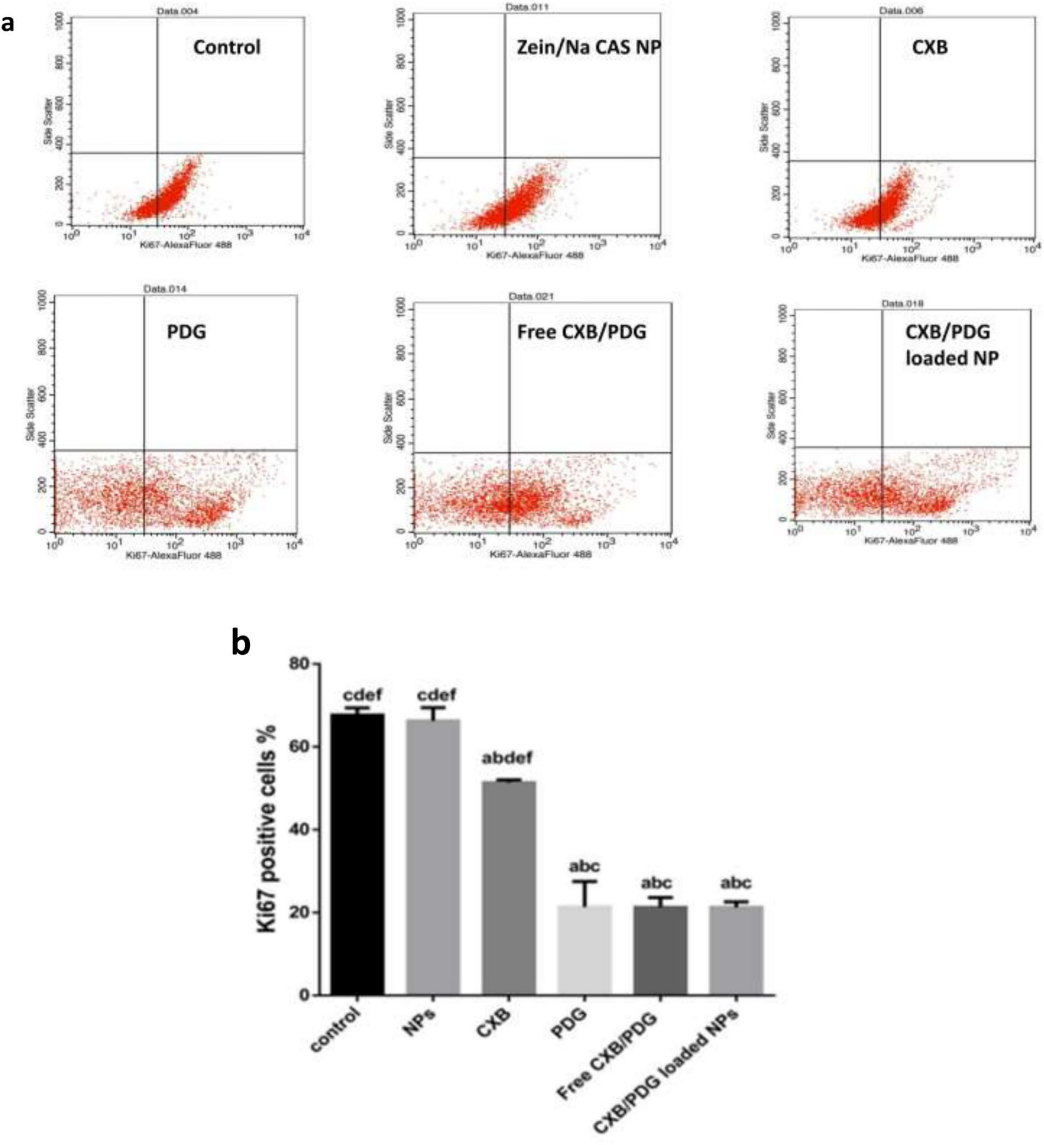
Dot plots of ki-67 antibody analysis of control, zein/Na CAS NPs, and 10 μg of free celecoxib (CXB), free prodigiosin (PDG), free CXB/PDG and CXB/PDG-loaded NPs-treated MDA-MB-231 cell line (**a**). Results were normalized according to the negative control. Relative change in ki-67 positive cells measured by flow cytometry (**b**). Values were represented as mean ± SD and n = 3. Statistical analyses were done using one-way ANOVA followed by Post Hoc Test (Tukey). (**a**) P ≤ 0.05 vs control, (**b**) P ≤ 0.05 vs zein/Na CAS NPs, (**c**) P ≤ 0.05 vs free CXB, (**d**) P ≤ 0.05 vs free PDG, (**e**) P ≤ 0.05 vs free CXB/PDG and (**f**) P ≤ 0.05 vs CXB/PDG-loaded NPs.

Results manifested that there was a significant difference in Ki-67 positive cells in all treated group except for zein/Na CAS NPs (67.645 ± 1.20%) which was comparable with the control untreated group (66.26 ± 2.30%). The % of Ki-67 in cells treated with free PDG, free CXB/PDG and CXB/PDG-loaded NPs were 21.43 ± 4.37%, 21.27 ± 1.67% and 21.285 ± 0.90%, respectively, which were significantly reduced in comparison to cells treated with free CXB which displayed about 51.4 ± 0.50% (Fig. 8a, b).

#### Effect of CXB, PDG and their combination on COX-2 expression level

**Figure 9:**
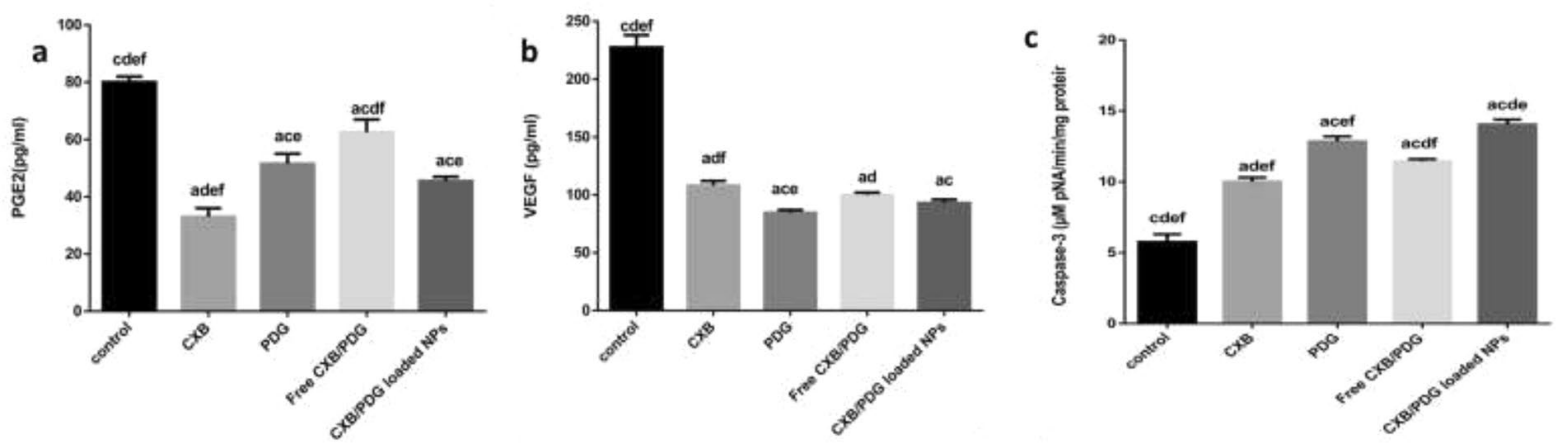
Prostaglandin E2 (PGE2) in cell culture (**a**), vascular endothelial growth factor (VEGF) in cell culture (**b**) and caspase 3 activity in cell lysate in treated and untreated group (**c**). Data were expressed as mean ± SD and n = 3. Statistical analyses were done using one-way ANOVA followed by Post Hoc Test (Tukey). (**a**) P ≤ 0.05 vs control, (**c**) P ≤ 0.05 vs free CXB, (**d**) P ≤ 0.05 vs free PDG, (**e**) P ≤ 0.05 vs free CXB/PDG and f P ≤ 0.05 vs CXB/PDG-loaded NPs.

As shown in Fig. 9a, there was an obvious inhibitory effect on COX-2 when cells were treated with free CXB, free PDG, free CXB/PDG and CXB/PDG-loaded NPs with PGE2 concentration of about 33 ± 3.0 pg mL^−1^, 51.5 ± 3.5 pg mL^−1^, 62.5 ± 4.5 pg mL^−1^ and 45.5 ± 1.5 pg mL^−1^, respectively compared to 80 ± 2.0 pg mL^−1^ for positive control. Most significant inhibition was in CXB treated group followed by CXB/PDG-loaded NPs treated group which exhibited an enhanced anti-inflammatory effect when compared to free PDG or free CXB/PDG.

#### Effect of CXB, PDG and their combination on VEGF expression level

As shown in (Fig. 9b), the most significant inhibition of VEGF level was observed in PDG-treated group which was 84.5 ± 2.5 pg mL^−1^, followed by CXB/PDG-loaded NPs (93 ± 3 pg mL^−1^), free CXB/PDG (100 ± 2 pg mL^−1^) and free CXB (108 ± 4 pg mL^−1^) compared to untreated control group which exhibited about 227.5 ± 10.5 pg mL^−1^. The inhibition effect of CXB/PDG-loaded NPs was more than free CXP/PDG, which might be due to the enhanced cellular uptake and increasing bioavailability of drugs inside cancer cells.

#### Effect of CXB, PDG and their combination on apoptosis

Activity of CAS 3 was measured as function of the amount of p-Nitroaniline (pNA) after hydrolysis of p-nitroanilide by caspase 3. As shown in Fig. 9c, there was a significant increase (p < 0.0001) in caspase 3 activity in free CXB, free PDG and free CXP/PDG-treated group which exhibited concentrations of about 10 ± 0.30, 12.85 ± 0.35, 11.45 ± 0.15 µM pNA min^−1^ mg protein^−1^, respectively, compared to control group which displayed activity of about 5.75 ± 0.55 µM pNA min^−1^ mg protein^−1^. Combination of CXB and PDG in their free form or their co-encapsulation in zein/Na CAS NPs resulted in enhanced the anticancer effect of both drugs. The most significant increase in the activity of the apoptotic biomarker; caspase 3 was in case of CXB/PDG-loaded NPs treated group (14.5 ± 0.35 µM pNA min^−1^ mg protein^−1^).

## Discussion

Peanut media provide maximum production amount of prodigiosin as it contains large amount of saturated fatty acids which plays a vital role in hyperpigmentation of *S. marcescens.* Beside that peanut powder is costless growth media. PDG color was found to change according to the pH of the solvent. This reversible shift in color might occur due to the formation of Na salt with nitrogen atom of pyrrole ring of the pigment and it depends also on [H^+^] concentration^16,18,66,67^. From the obtained result, it can be concluded that the extracted pigment is prodigiosin.

Dual drug-loaded zein NPs were prepared by antisolvent method in which; CXB, PDG and zein were co-dissolved in 85% ethanol and dropped into water and while evaporation of ethanol, hydrophobic zein will be changed into zein NPs but with low stability especially in long term storage^32^. Consequently, zein NPs can be coated by another natural biopolymer like pectin or caseinate to increase their colloidal stability. However, caseinate was found to be better than pectin as pectin coatings usually yields larger particle size^52^. The prepared NPs showed good PS with zeta and sustained release because of their encapsulation into the hydrophobic core of the NPs and high miscibility of both drugs with the core of zein NPs due to their high hydrophobicity as PDG and CXB have log P values of about 5.16^68^ and 3.5^61^, respectively.

The most fitting release kinetic model for CXB and PDG in PBS supplemented with 50% ethanol release media at different pH was the first order kinetic model which depends on Noyes-Whitney equation which refers to dissolution of drugs that might occur during release process (actual surface of material cannot be constant during dissolution)^69^. The good linearity obtained from the results could be attributed to dependency of drug release rate on the concentration of drug within the core of the NPs, which means that the amount of released drug will decrease by reducing its concentration. In other words, release media will diffuse through matrix and forces drug to be released^70^.

Regarding release profile of CXB in PBS; pH 7.4, the kinetic model which fitted release profile of CXB in PBS at pH 7.4 was Higuchi model which depends mainly on diffusion of the encapsulated drug from the inner core of NPs into release media^71^. Moreover, other factors might affect this model including; amount of the drug inside NPs is higher than its solubility, drug diffusion might occur in one dimension, particles of the drug are much smaller than thickness of the carrier or the swelling or dissolution of the nanocarrier is weak^72^. From these results, it can be concluded that presence of ethanol in the release media altered the kinetic behavior of the drug release. In case of PBS only, CXB was able diffuse from NPs into the release media, but PDG was not able to do so, might be due to its high lipophilicity. In case of media supplemented with ethanol, the media was able to diffuse into the inside of the NPs causing release of both CXB and PDG as shown in supplementary file.

Hemocompatibility of NPs is an important issue for developing a biocompatible nanocarrier for drug delivery purposes. When NPs are administrated intravenously, they interact with blood protein and form protein corona. Consequently, interaction between blood components and NPs must be taken into consideration. There are multiple factors which might affect the interaction between NPs and RBCs causing blood hemolysis. These factors include; charge, size, shape, and composition of NPs. For instance, positively charged NPs have a high tendency for electrostatic interaction with negatively charged RBCs membrane leading to release of hemoglobin from erythrocytes^73–75^. The resulted Hemolysis % ware less than (5%) which considered as safe and prove hemocompatibility of the formulated NPs^56^. The prepared nanoformulation has no effect on RBCs due to the negative charges on the surface of CXB/PDG-loaded NPs which caused repulsion with negatively charged RBCs^76^. In addition, Hydrophilic nature of Na CAS shell which surround zein NPs also participate in reducing RBCs hemolysis of the resultant formulation^56^.

From cytotoxicity results, the enhanced cytotoxic effect of dual drug-loaded NPs might be related to the ability of Na CAS outer shell to improve stability and enhance cellular uptake of zein NPs via increasing the amount of the negative charge, which as a result might improve the interaction with the cancerous cell membrane^74^. The superiority of CXB/PDG-loaded NPs in reducing viable cell % and induction of apoptosis could be due to enhanced cellular uptake of NPs. For instance, NPs with size less than 200 nm are supposed to be uptaken by cells via clathrin-mediated endocytosis, besides enhanced permeability and retention effect (EPR), while NPs with size more than 200 nm will enter the cells by caveolae-mediated pathway^77^. Moreover, positively charged NPs have higher cellular uptake in comparison to neutral and negatively charged NPs. Enhanced cytotoxic effect of CXB/PDG-loaded NPs is due to adsorption of caseinate onto the surface of zein NPs which might decrease interfacial tension leading to enhanced interaction of zein NPs with the cell membrane and increased its cellular uptake^76,78^. Although the obtained results from the MTT assay suggested that the anticancer effect of each drug was at different ratio. However, when the cells were treated with drug-loaded NPs, the DL% ratio 1:1, from PDG: CXB, not 1:4. This was done to compensate the slow release of both drugs; especially PDG from the nano-formulation. In addition, in vitro released studies showed that CXB was released at higher rate in comparison to PDG, which suggests the concentration of liberated CXB will usually be higher than that of PDG.

Metastasis is an important factor in cancer progression. In general, cellular migration is a multistep process including; protein degradation of extracellular matrix, cancer cells’ entry into blood circulation, followed by their migration through blood stream and finally adherence into other secondary organs^79^. Both CXB and PDG are known to inhibit Wnt/β-Catenin and PI3K/AKT pathways which have a vital role in several processes such as proliferation, migration, cell survival and self-renewal of cancer stem cell^24,58,80^. The most detected inhibition of cancer cell migration was observed when cells were treated with free PDG, free CXB/PDG and CXB/PDG-loaded NPs group. Although, the inhibition of migration by CXB/PDG-loaded NPs has the most pronounced effect but not significantly different from free PDG, which proved that nanoencapsulation of PDG does not effect on its antimigratory effect which be attributed to the sustained release of both drugs from the core of the NPs and this inhibition is expected to increase if it was incubated for a longer time^81^. The enhanced inhibition of migration might be obtained by small size of the resulted NPs and increased cellular uptake^78^. Chunbai et al.^82^. reported that negative charge on the surface NPs which have size ~ 150 nm tends to be accumulated inside tumor cell.

The prominent proliferative marker: Ki-67 (proliferating cell nuclear antigen PCNA) is usually related to cell proliferation and transcription of the RNA^83^. This nuclear protein is usually associated with poor prognosis and low survival rate. Ki-67 is an important therapeutic target for cancer, as it is overexpressed in malignant cells but rarely detected in normal cells, and hence its expression is commonly used as a prognostic marker to predict the response of some patients towards specific treatments^84–86^. Celecoxib was reported to decrease Ki-67 expression and prostaglandin E2 production in heavy smokers and reduce lung cancer risk^87^. Therefore, CXB may exert its anti-proliferative effect in breast cancer cells via both COX-2 dependent and COX-2 independent pathways^9^. Furthermore, PDG was also reported to inhibit growth and proliferation of tumor cells^24^. From our result, there is a significant reduction in Ki-67 expressing cells when they were treated with PDG, free combination and formulated NPs. The reduction in Ki-67 expressing cells may be due to the repression of tumor growth and stimulation of apoptosis. However, CXB was found to be more effective when it is used as an adjuvant with other anticancer agents such as PDG, otherwise, it might require a higher dose to exert an anticancer effect solely^58^. CXB has no effect on proliferation of tumor cell, according to several studies. Even if it inhibits proliferation, it will do so in a time and dose dependent manner^88^. These results suggested that CXB is a potent COX-2 inhibitor, but it can’t exert an inhibitory effect on cancer cell proliferation especially at the low utilized dose.

Inflammation is considered as a hall marks of cancer progression. PGE2 (key mediator of inflammation), plays a vital role in tumor development, invasion, inhibition of apoptosis, metastasis, angiogenesis and modulation of antitumor immunity^89^. PGE2 first binds to a G-protein coupled receptors (GPCRs) called EP and divided into EP-1, EP-2, EP-3, and EP-4 subclass. The biological function of PGE2 depends on the stimulation of specific EP receptor. EP receptors influence cell response to PGE2 mainly via EP1-dependent migration and invasion, EP2-induced angiogenesis, EP4-related migration, and EP3-mediated carcinogenesis. PGE2 was synthesized by COX-2 is physiologically absent in normal cells and highly expressed in different types of carcinoma^90^. As a result, inhibition of PGE2 production by inhibiting COX-2 can lead to down regulation of Wnt pathway, and hence might inhibit the stemness properties of breast cancer cells. Additionally, PDG also is reported to be an effective Wnt/β-Catenin pathway inhibitor by targeting several sites^24^. CXB exerts its anticancer effect by inhibiting tumor initiation, proliferation, chemo-resistance of cancer cells and recurrence^58^. An in silico anti-inflammatory study of PDG against CXB as COX-2 inhibitor revealed that PDG can interact with two amino acids; Leu321 and Tyr324 in the active site of COX-2 and has the highest fitness score compared to CXB, so it is supposed for PDG to act as an anti-inflammatory effect, too^26^. From the current study, the anti-inflammatory effect of PDG was found to be less than that of CXB. The most significant inhibition of COX-2 was observed in cells were treated with CXB alone followed by the formulated NPs treated cells. The enhanced anti-inflammatory effect of the prepared NPs might be due to their enhanced cellular uptake^82^.

Vascular endothelial growth factor is an important angiogenic factor included in tumor angiogenesis and it is upregulated in many different types of tumors. CXB was reported to inhibit VEGF by hindering the binding between DNA and SP1 protein which is critically associated with VEGF expression. COX-2 inhibition by CXB was also reported to reduce VEGF expression level^91^. On the other hand, PDG was previously reported to directly inhibit VEGF level in MDA-MB-231 cell line. Previous studies reported that, there was possible cross talk between MMP-9, VEGF, and Ki-67^92^. From our result, PDG was significantly inhibits VEGF expression level which might explain why it significantly inhibits Ki-67 expressing cells.

Apoptosis can be triggered in tumor cells by either intrinsic or extrinsic pathways. The intrinsic pathway can be initiated by permeabilization of the outer membrane of mitochondria followed by release of cytochrome C, with apoptosome formation resulting in caspases activation. Extrinsic pathway needs an external stimuli such as hypoxia or stroma to induce specific receptors on the cell membrane to activate adaptor proteins include; fas-associated protein with death domain (FADD) and caspase 8 which can cause permeabilization of mitochondrial membrane and control release of cytochrome C like intrinsic pathway^93^. Caspases are cysteine-aspartate proteases that are essential to mediate programed cell death. Caspase 3 is considered as a significant molecule for induction of either extrinsic or intrinsic apoptotic pathway and its activity is mostly measured as an indicator for cancer cell death^94^.

Prodigiosin is known to intercalate with DNA and inhibit topoisomerase I and II in acute human T leukemia and MCF-7 cell lines^20^. Wnt pathway could be a significant target in triple negative breast cancer (TNBC) which is a subtype of breast cancer that lacks efficient therapeutic approaches^95^. Hyperactivation of Wnt signal pathway leads to translocation and accumulation of β-Catenin inside the nucleus which can then serve as a transcription factor for expression of target genes like cyclin D1 causing tumor growth and proliferation^80^. Dysregulation of Wnt pathway is usually found in tumor cells and inhibition of this pathway might induce apoptosis, inhibit proliferation, migration and cell survival^14^. PDG was also found to decrease the antiapoptotic protein; survivin and induce the pro-apoptotic protein; P53, which can cause cell cycle arrest and induction of apoptosis by increasing caspase 3 expression in acute lymphoblastic leukemia cells^96^. PDG also exerts its anticancer effect and overcome drug resistance in TNBC by inhibiting PI3K/AKT/mTOR via decreasing the expression level of m-TOR^25^. PDG and 5-FU -loaded probiotic bacterial ghost was found to induce apoptosis in colon cancer cell line via increasing caspase 3 expression level^62^. Moreover, PDG has a unique feature as it is not a substrate for multi-drug resistance proteins like breast cancer resistance protein (BCRP) and multi drug resistance pumps (MDR) with no toxic effect on normal cells. It can also induce mitochondrial mediated apoptosis in MCF-7 and MDA-MB-231 breast cancer cell lines regardless presence or absence of ABC transporter^97,98^.

Furthermore, CXB treatment was found to decrease the proliferation of estrogen receptors MCF-7 and MDA-MB-231cells. CXB can induce a significant suppression in COX-2 levels in MDA-MB-231 cells, associated with a decline in aromatase expression and proliferation of tumor cells. So, CXB may exert its antiproliferative effect via induction of apoptosis in breast cancer cells by both COX-2 dependent and COX-2 independent pathways as previously reported^9^. Combination of CXB and PDG in their free form or their simultaneous encapsulation in zein/Na CAS NPs enhanced the anticancer effect of both drugs. This enhancement was revealed by significant increase in the activity of the apoptotic biomarker; caspase 3 in case of CXB/PDG-loaded NPs-treated group which might be related to better cellular internalization of the formulated NPs^78^.

## Conclusion

Zein and sodium caseinate biopolymers can be used to fabricate polymeric NPs instead of synthetic polymers owing to their biocompatibility and minimal toxicity. In this study, zein/Na CAS NPs were fabricated and co-encapsulated with CXB and PDG using a facile preparation technique. CXB/PDG-loaded NPs showed reduced hemolytic rate with particle size of about 171.2 ± 3.535 nm and zeta potential of about − 34.9 mV. The superior antitumor activity of CXB/PDG-loaded NPs on MDA-MB-231was observed by the reduction in the combination index of free CXB/PDG from 0.62251 to 0.15493 in case of their, suggesting a possible synergism between both drugs. Furthermore, CXB/PDG-loaded NPs were found to induce apoptosis with increased caspase 3 activity, inhibit proliferation with reduced ki-67 expression level, besides their potential to inhibit cellular migration and angiogenesis. Collectively, this improved in vitro antitumor efficacy might be related to enhanced stability of the negatively charged NPs, better cellular internalization, and hence enhanced accumulation and bioavailability of drugs inside cancer cells.

## Materials and methods

### Materials

Celecoxib was kindly obtained as a gift from (El Borg pharmaceutical industries, Egypt). PDG was extracted from *Serratia marcescens.* Zein, sodium caseinate, DMSO, methanol HPLC grade and CASP-3-C ELISA kit were purchased from (Sigma Aldrich Inc., USA). Dulbecco's modified Eagle's medium (DMEM), Pierce BCA protein assay kit, Alexa Fluor® 488 and Anti-Ki67 antibody were purchased from (Thermofisher Scientific Inc., USA). Penicillin/Streptomycin Solution (100x) and fetal Bovine Serum (FBS) were purchased from (Cegrogen Biotech, Germany). RIPA2 lysis buffer was purchased from (BOSTER BIO Inc., China). Human VEGF antibody kit Quatikine ELISA kit was purchased from (R&D technology Inc., USA). Human prostaglandin E2 ELISA kit was purchased from (CUSABIO Inc., China). All other utilized reagents were from analytical grade, and they were utilized without any modification.

### Production and purification of PDG from *S. marcescens*

*Serratia marcescens* (a prodigiosin producing strain) was previously characterized and identified^25^. *S. marcescens* was activated in 50 mL of preculture medium (10 g peptone, 10 g yeast extract, 2 g K_2_HPO_4_ and 10 g dextrose per 1 L distilled water) in an Erlenmeyer flask and it was incubated in a shaking incubator (shaking incubator, SI-100R, HUMAN lab, France) at 150 rpm and 28 °C for 18 h. To enhance prodigiosin production, 5 mL of overnight *S. marcescens* culture was inoculated on 100 mL of 2% peanut medium, pH 7. The inoculated medium was incubated in a shaking incubator at 180 rpm and 28 °C for 48 h in a 1L Erlenmeyer flask^16^. Extraction was done according to Hubbard and Rimington^18^ with some modifications^18^. In brief, 10% NaOH was added to the culture (100 mL) and incubated in dark for 2 h, then equal volume of absolute ethanol was added and incubated while shaking at 120 rpm for another 2 h. After that, equal volume of petroleum ether 60–80% was added fraction by fraction in a separating funnel and it was allowed to stand for a while to be separated into two layers; upper organic layer containing the pigment and lower aqueous layer which was re-extracted again. Finally, the obtained pigment dissolved in the organic solvent was dried in oven at 37 °C and stored at – 20 °C till further utilized. Purification of PDG was performed by using silica gel C18 reversed phase column which yield a good pigment purity according to Anwar et al.^25,42^.

### Characterization of prodigiosin

#### UV–visible spectroscopy

Prodigiosin was first dissolved in absolute methanol, then the pH was adjusted to acidic range (pH = 2) using 1M HCl, basic range (pH = 9) using 1% NaOH and it was also kept at neutral pH, then the three preparations were scanned using a UV–visible spectrophotometer (Thermo scientific®, Evolution 300, USA) at wavelength range from 400 to 600 nm with methanol as the blank^99^.

#### Thin layer chromatography (TLC)

Purified PDG was dissolved in methanol. Mobile phase was composed of methanol: ethyl acetate: chloroform (6: 3:1; v/v/v). The mobile phase (10 mL) was put in a tight closed glass jar and left till saturation. Sample was then spotted on TLC plate and allowed to be developed^100^.

#### FTIR analysis

Spectrum of PDG was performed using RXI FTIR spectrometer (Perkin Elmer, USA). The sample was mixed well with KBr (IR grade), then the spectrum was recorded in the transmission mode at an ambient temperature over the range of 4000–400 cm^-1^^56^.

#### Characterization of PDG by HPLC

Prodigiosin (5 mg mL^−1^) was dissolved in acidified methanol, then 20 μL of PDG stock solution was injected into YL9100 HPLC system (YOUNG LIN, Korea). The HPLC YL 9101 Vacuum degasser system was supplied with a Premosil C18 column (5 μm, 100 Å, 4.6*150 mm). Mobile phase was methanol: ammonium acetate buffer; pH 3 (90:10; v/v) with flow rate 2 mL min^−1^ and run time about 15 min^42,101^.

#### Preparation of CXB/PDG-loaded zein/Na CAS NPs

Dual drug-loaded Zein/Na CAS NPs were prepared by antisolvent technique as previously reported^102^. In brief, 5 mg of both CXB and PDG were dissolved in 4.2 mL of absolute ethanol for 1 h at room temperature and 800 rpm (Wise Stir, MSH-30D, Korea), then 0.8 mL of distilled water was added, followed by addition of 50 mg zein under magnetic stirring for 1 h at 800 rpm. In another beaker, 50 mg of Na CAS was dissolved in 15 mL of distilled water at 500 rpm for 2 h. Afterwards, zein and the drugs mixture solution was rapidly added onto Na CAS solution and the speed of stirring was increased to 1200 rpm and it was left for 3h to ensure complete ethanol evaporation and formation of CXB/PDG-loaded NPs. Blank zein/ Na CAS NPs was prepared as the abovementioned method but without drugs addition. Finally, the dual-loaded NPs solution was stored for further characterization.

### Physicochemical characterization of PDG/CXB-loaded zein/NA CAS NPs

#### FTIR spectroscopy

Spectra of CXB, PDG, zein, Na CAS, zein/Na CAS NPs and CXB/PDG-loaded NPs were recorded using RXI FTIR spectrometer (Perkin Elmer, USA).

#### DSC analysis

Thermograms of free CXB, zein/Na CAS NPs and CXB/PDG-loaded zein/Na CAS NPs were recorded by using (STD650, TA instruments, USA). About 2 mg of each sample were put in a sealed aluminum pan, then it was heated at 10 °C min^−1^ under nitrogen atmosphere with flow rate of 20 mL min^−1^ (heat range 25–400 °C). An empty aluminum pan was included as reference^31^.

#### Particle size (PS), polydispersity index (PDI) and zeta potential

The PS of both blank and dual-loaded NPs were measured by using Nano-ZS/ ZEN3600 zeta sizer (Malvern Instruments Ltd., UK). Photon correlation spectroscopy (PCS) was used to quantify (PDI) and (PS) using non-invasive backscattering equipment, the PS was measured at a 173° detection angle after dilution of each sample to proper concentration using double filtered distilled water. Triplicates of each DLS measurement were performed at an ambient temperature. In a universal folding capillary tube with platinum electrodes, each sample was diluted by double filtered water for zeta potential measurements. Laser Doppler Anemometry (LDA) calculation of the mean electrophoretic mobility was used to measure zeta potential values^79^.

#### Morphological examination by TEM

Freshly prepared CXB/PDG-loaded NPs were visualized by JEM-1400 Plus electron microscope (JOEL, Tokyo, Japan) with accelerating voltage of 80 kV and magnification power 25 K. NPs solution was diluted at appropriate ratio with purified deionized water and sonicated for 10 min, followed by staining with uranyl acetate and spotting on a copper grid^31^.

#### Encapsulation efficiency and drug loading %

The EE% of CXB and PDG in NPs were measured directly by centrifugation of 1 mL of the formula at 12,000 rpm for 1 h (Hettich Mikro 120 centrifuge, AB Lab Mart, Malaysia), then the pellet was dissolved in 5 mL absolute ethanol, and the drugs concentrations were measured by YL9100 HPLC system (YOUNG LIN, Korea). For simultaneous determination of both CXB and PDG, a new HPLC method was developed with mobile phase 90:10 (% v/v; methanol: acidified water, pH3. The injection volume was 20 μL with flow rate 1.5 mL min^−1^. The HPLC YL 9101 Vacuum degasser system was supplied with a Premosil C18 column (5 μm, 100 Å, 4.6*150 mm). CXB was detected at 250 nm and PDG at 530 nm with a total run time of 6 min. The EE% and DL % were measured as follows:

1 $${\text{EE}}\% \, = \frac{Mass \;of\; encapsulated\; drug\; in \;NPs}{{Initial\; mass of\; drug\; added}} \times 100$$

2 $${\text{DL}}\% \, = \frac{Mass \;of\; encapsulated \;drug}{{Mass \;of \;encapsulated\; drugs + mass\; of\; NPs }} \times 100$$

#### In vitro drug release study

The release profiles of both free and-loaded drugs were determined using dialysis bag method in three different release media including; PBS at pH 7.4, PBS: 50% ethanol at pH 7.4 and PBS: 50% ethanol at pH 5.4. 2mg of each free drug or an equivalent amount of drug-loaded NPs were added into a presoaked dialysis bag (12–14 k Da MWCO VISKING dialysis tubing, SERVA, Germany). After wards, the dialysis bag was immersed in 200 mL release media and incubated in a shaking water bath (DKZ series shaking water bath, AZZOTA Corporation, Zhejiang, China) at 100 rpm and 37°C. Two mL of each release medium was withdrawn at certain time interval and compensated with 2 mL of fresh medium, then the amount of the released drug was estimated using HPLC and calculated as follows:

3 $${\text{Cumulative }}\;{\text{drug }}\;{\text{released }}\% = \frac{Amount \;of \;released \;drug\; at\; time}{{Total \;amount \;of \;encapsulated \;drug}}*{1}00\%$$

The model of drugs release from prepared nanoformulation was determined by analyzing the results of in vitro release experiment with model-dependent methods. The drug release kinetic models including; first-order kinetic, zero-order kinetic, Higuchi kinetic, and Korsmeyer-Peppas kinetic models were used in data analysis. The determination of suitable kinetic model based on better linearity in terms of correlation coefficient (R^2^). In zero order kinetic model, data obtained from the release study were plotted as cumulative drug release % (%CDR) versus time (h). In this model, the rate of released drug was found to be independent on drug concentration. In first order kinetic model, data obtained from the release study were plotted as log cumulative drug remaining within NPs versus time (h). In Higuchi kinetic model, data obtained from the release study were plotted as %CDR versus square root of time (h), while in Korsmeyer-Peppas model, data of the release study were plotted as log %CDR versus log time (h)^69^.

#### In vitro blood hemolysis

Released hemoglobin from red blood cells (RBCs) was determined after incubation of CXB/PDG-loaded NPs with erythrocytes as previously reported with some modifications^31^. Fresh blood from a healthy Swiss albino mouse was collected on EDTA-coated test tubes. The collected blood was then centrifuged at 3000 rpm (TJ-6 centrifuge, BECHMAN, USA) for 5 min. Afterwards, the clear plasma layer was discarded, and the RBCs pellet were rinsed twice with saline. Red blood cells were diluted (1:10, v/v) with sterile saline, then 2 mL of diluted RBCs suspension were mixed with an equivalent volume of CXB/PDG-loaded NPs (0.5 and 1 mg mL^−1^) and incubated with gentle shaking at 37 °C for 1h. A blank was prepared for each concentration by dissolving an equivalent amount of dual drug-loaded NPs in saline as PDG was measured spectrophotometrically at 535 nm^101^. After incubation, the integrity and morphology of RBCs were examined by a light microscope (Zeiss Primo star microscope, Germany) and the amount of released hemoglobin was determined by spectrophotometer at 545 nm according to the following equation. Negative control was prepared by addition of 2 mL of diluted RBCs solution with 2 mL saline (0% lysis). For positive control, saline was replaced with water (100% lysis).

4 $$\% {\text{ Hemolysis }} = \frac{{A_{s} - A_{nc} }}{{A_{pc} - A_{nc} }}*100\%$$

A_s_: absorbance of the sample, A_nc_ = absorbance of negative control., A_pc_ = absorbance of positive control.

### In vitro anticancer efficacy

#### In vitro cytotoxicity study

The cytotoxic effect of free drugs, blank NPs and dual-loaded NPs were assessed by MTT (3-[4,5-dimethylthiazol-2-yl]-2,5 diphenyl tetrazolium bromide) assay depending on the conversion of MTT into formazan crystals via mitochondrial activity of viable cells^103^. Human triple negative breast cancer MDA-MB-231 cells were purchased from the American Type Culture Collection (ATCC), USA. Cells were seeded with concentration of about 7*10^3^ cells well^−1^ in 96 well microtiter plate and incubated in CO_2_-incubator (Thermofisher, USA) at 37 °C and 5% CO_2_ for 24 h till reaching 80% confluency. Afterwards, the media in each well was replaced by 100 μL of culture media containing CXB, PDG, free drugs combination, blank NPs, and dual drug-loaded NPs in concentration range (0.1–20 μg mL^−1^), followed by incubation for 72 h at the same conditions. Stock solutions of CXB and PDG were prepared in DMSO (5 mg mL^−1^) individually and free combined drugs were also prepared in DMSO (5mg CXB + 5mg PDG in 1 mL). DMSO concentration in culture media was kept at less than 0.6% which was proven to be safe for cells^104^. Media containing drugs was replaced with 20 μL MTT solution (5 mg mL^−1^) and incubated for further 4 h under light protection. Media was discarded then replaced again with 150 μL DMSO and left for 15 min while shaking at 100 rpm in order to solubilize formazan crystals. Absorbance of solubilized formazan crystals was determined at 570 nm and 690 nm for background subtraction by microplate reader (Tecan, USA). Cell viability of treated groups was expressed as a percentage in comparison to the control group^31^. The IC_50_ of (CXB & PDG) and the combination indices of (free combined drug & dual drug-loaded NPs) were calculated by COMPUSYN software version 1.

#### Migration assay

Migration of breast cancer cells was assessed by scratch wound healing assay. Briefly, cancer cells were grown in 6-well plates for 24 h till confluency reached 80%. After that, a sterile tip was used to make a wound in the cellular monolayer. Cells were then treated with zein/Na CAS NPs (equivalent amount to CXB/PDG-loaded NPs), 10 μg mL^−1^ of CXB, PDG, free CXB/PDG or CXB/PDG-loaded NPs for 24 h. Images were taken using the light microscope at zero time and after 24 h of treatment to monitor the wound closure. Image J software (NIH, Bethesda, MD) was used to analyze and measure width of the wounds before and after treatment. Wound closure % was expressed relative to wound size before treatment as follows^105^:

5 $${\text{Wound}}\;{\text{ closure }}\% \, = \frac{{W_{i} - W_{f} }}{{W_{i} }}*100\%$$

W_i_: initial width of the wound, W_f_: final width of the wound.

#### Tumor growth biomarkers

Tumor cells were seeded in 6-well plates at a density of 3 × 10^5^ cells well^−1^ and they were allowed to grow for 24 h till confluency reached 80%. After that, culture media was replaced by fresh media including 10 μg of CXB, PDG, free CXB/PDG, CXB/PDG-loaded NPs or equivalent amount of zein/Na CAS NPs, followed by incubation for 72 h. After that, Ki-67 was measured as a biomarker for proliferation by Alexa Fluor® 488 Anti-Ki67 antibody using flow cytometry (Thermofisher Scientific Inc., USA). The negative control was cells without antibody which was used due to PDG autofluorescence with excitation at 543 nm and an emission at 570 which is within the range of the labeled antibody^106^. As an apoptotic marker, Caspase 3 activity was determined in cell lysate by CASP-3-C ELISA kit (Sigma Aldrich Inc., USA) according to the manufacturer’s protocol. Cell lysate was prepared from cell pellets via hydrolysis using RIPA2 lysis buffer (BOSTER BIO Inc., China). Total protein concentration was determined in cell lysate by Pierce BCA protein assay kit (Thermofisher Scientific Inc., USA). Human vascular endothelial growth factor (VEGF) and expression of COX-2 was measured as a function of human prostaglandin E2 (PGE2) were measured by Quatikine ELISA kit (R&D technology Inc., USA) and human prostaglandin E2 ELISA kit (CUSABIO Inc., China), respectively according to the manufacturers’ protocol.

### Statistical analysis

Statistical analyses were conducted by using Graph Pad Prism version 6 software. Analysis of Variance was done by one way ANOVA followed by Tukey’s Multiple Comparison (TMC) for pairwise comparisons between the different groups. The significance of the resulting data was assessed at 5% level.

### Ethical approval

Animal experiment was approved by ethics committee of the institute of graduate studies and research with the Alexandria university ethics code (AU14-23061-1-2). All authors complied with the arrive guidelines and all experiments were performed according to guideline and regulations. All authors consent to participate in this manuscript. All the authors consent the publication of the manuscript. All authors declare that no competing interests related to the work.

### Supplementary Information

Supplementary Information.

## Data Availability

All data in this study are publically available and the raw analysis data can be obtained by contacting the corresponding author upon request.
