# Supplementary material for: Prodigiosin/celecoxib-loaded into zein/sodium caseinate nanoparticles as a potential therapy for triple negative breast cancer
Source: Sci Rep. 2024 Jan 2;14:181. doi: 10.1038/s41598-023-50531-4 (PMC10761898; doi:10.1038/s41598-023-50531-4)
Supplement: Supplementary file 1 — Supplementary Information. [file 41598_2023_50531_MOESM1_ESM.docx]

**Supplementary file**

Supplementary figure 1 (S1): Particle size of celecoxib/ prodigiosin loaded zein/ Na caseinate Nps at PBS; pH 7.4 and 50% ethanol (**a**) and PBS; pH 5.4 and 50% ethanol (**b**).


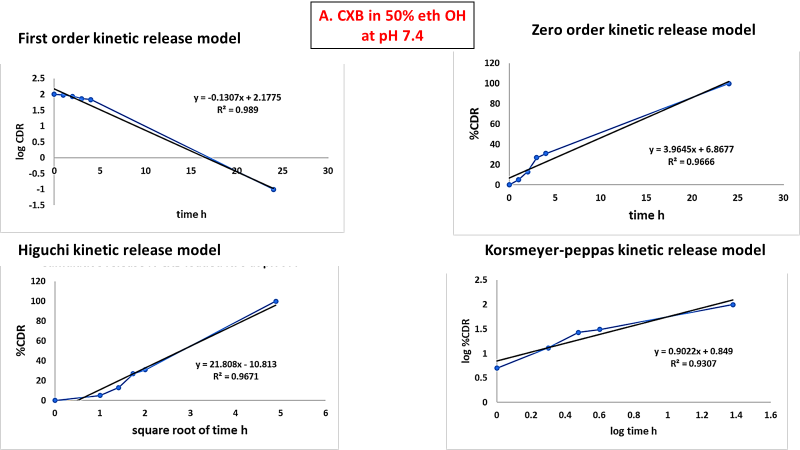

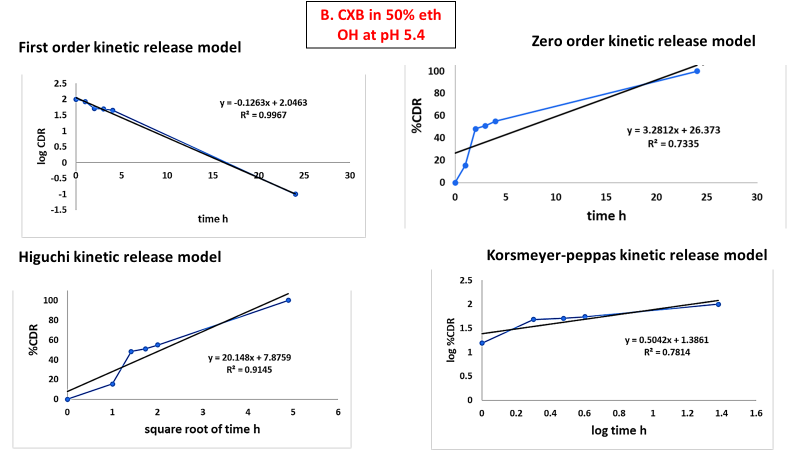

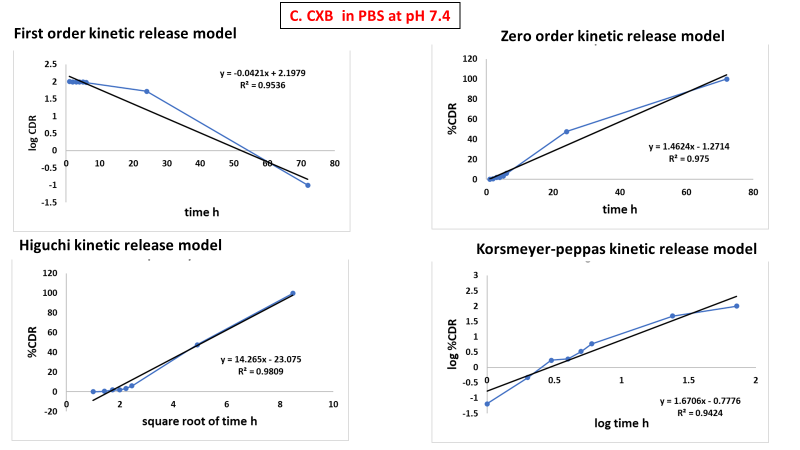


Supplementary figure 2 (S2): Different Kinetic release curves of celecoxib( CXB) at (**a**) PBS; pH 7.4 and 50% ethanol, (**b**) PBS; pH 5.4 and 50% ethanol and (**c**) PBS at pH 7.4‎.


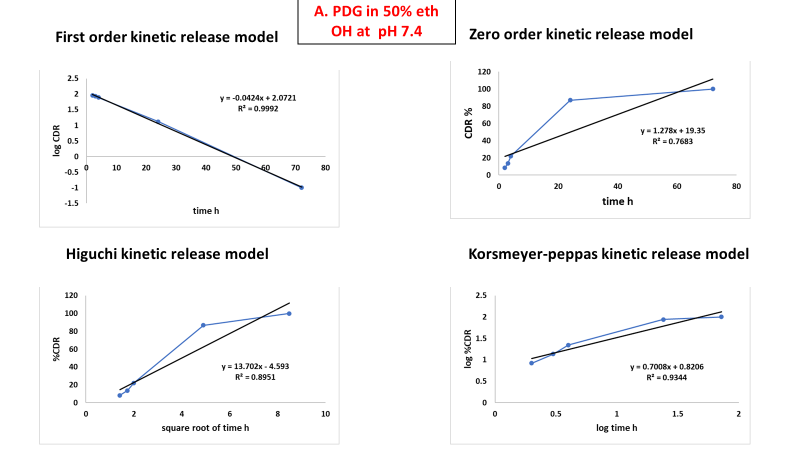

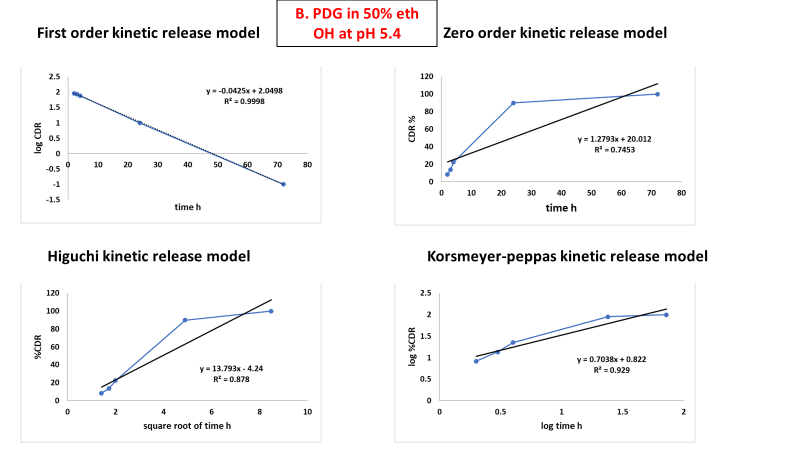


Supplementary figure 3 (S3): Different Kinetic release curves of prodigiosin (PDG) at (A) PBS; pH 7.4 and 50% ethanol, (B) PBS; pH 5.4 and 50% ethanol.

**Supplementary Table 1.** Different R^2^ obtained from different kinetic model fitting curves of CXB and PDG in different release media.

| R^2^ | CXB in PBS pH 7.4, 50% ethanol | PDG in  PBS pH ‎‎  7.4,50% ‎ethanol ‎ | CXB in PBS pH 5.4, 50% ‎‎ethanol | PDG in PBS pH 5.4, 50% ‎ethanol | CXB in ‎PBS pH 7.4‎ |
| --- | --- | --- | --- | --- | --- |
| First-order model | **0.989** | ‎**0.999**‎ | ‎**0.996‎** | ‎**0.999‎** | ‎0.935‎ |
| Zero-order model | 0.966 | ‎0.768‎ | ‎0.733‎ | ‎0.745‎ | ‎0.975‎ |
| Higuchi model | 0.967 | ‎0.895‎ | ‎0.914‎ | ‎0.878‎ | ‎**0.980‎** |
| Korsmeyer-Peppas model | 0.930 | ‎0.934‎ | ‎0.975‎ | ‎0.929‎ | ‎0.942‎ |
